# Pediatric thiamine deficiency disorders in high‐income countries between 2000 and 2020: a clinical reappraisal

**DOI:** 10.1111/nyas.14669

**Published:** 2021-07-26

**Authors:** Benjamin Rakotoambinina, Laurent Hiffler, Filomena Gomes

**Affiliations:** ^1^ Cellular Nutrition Research Group Lagny sur Marne France; ^2^ LRI Isotopic Medicine Physiology Lab University of Antananarivo Antananarivo Madagascar; ^3^ The New York Academy of Sciences New York New York; ^4^ NOVA Medical School Universidade NOVA de Lisboa Lisboa Portugal

**Keywords:** pediatric thiamine deficiency, hyperlactatemia, Wernicke encephalopathy, bariatric surgery, sweetened drinks, anorexia

## Abstract

Often thought to be a nutritional issue limited to low‐ and middle‐income countries (LMICs), pediatric thiamine deficiency (PTD) is perceived as being eradicated or anecdotal in high‐income countries (HICs). In HICs, classic beriberi cases in breastfed infants by thiamine‐deficient mothers living in disadvantaged socioeconomic conditions are thought to be rare. This study aims to assess PTD in HICs in the 21st century. Literature searches were conducted to identify case reports of PTD observed in HICs and published between 2000 and 2020. The analyzed variables were age, country, underlying conditions, clinical manifestations of PTD, and response to thiamine supplementation. One hundred and ten articles were identified, totaling 389 PTD cases that were classified into four age groups: neonates, infants, children, and adolescents. Eleven categories of PTD‐predisposing factors were identified, including genetic causes, lifestyle (diabetes, obesity, and excessive consumption of sweetened beverages), eating disorders, cancer, gastrointestinal disorders/surgeries, critical illness, and artificial nutrition. TD‐associated hyperlactatemia and Wernicke encephalopathy were the most frequent clinical manifestations. The circumstances surrounding PTD in HICs differ from classic PTD observed in LMICs and this study delineates its mutiple predisposing factors. Further studies are required to estimate its magnitude. Awareness is of utmost importance in clinical practice.

## Graphical abstract

Pediatric thiamine deficiency (PTD) is perceived as being eradicated or anecdotal in high‐income countries (HICs). This study aims to assess PTD in HICs in the 21st century. Literature searches were conducted to identify case reports of PTD observed in HICs and published between 2000 and 2020. The circumstances surrounding PTD in HICs differ from classic PTD observed in low‐ and middle‐income countries and this study delineates its mutiple predisposing factors.

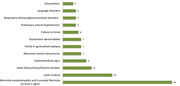

## Introduction

Thiamine, also called vitamin B1 or aneurin, is a water‐soluble vitamin that is an essential micronutrient for human beings, who cannot synthesize it and whose body reserves in tissues can be depleted within a couple of weeks without adequate thiamine intake.^1^ Thiamine is crucial factor for both enzymatic and nonenzymatic functions within the cytosolic, mitochondrial, and peroxisomal compartments. Thiamine is involved in many processes, particularly in energy transformation and oxidative and nonoxidative carbohydrate metabolism. As a crucial cofactor in the metabolism of carbohydrates and amino acids, its role is extended to fatty acid metabolism.^1^, ^2^ The limited body storage in tissues, combined with low intake, contributes to the propensity to thiamine deficiency (TD) during all life stages of human beings.

The daily recommended dietary allowance for thiamine has been revised based on the specific population requirements in some nations, which varies according to age range, gender, life stage (pregnancy and lactation), energy intake (notably carbohydrate intake), and physical activity level (inactive, moderately inactive, moderately active, and active occupational physical activity in children).^3^, ^4^ These recommended thiamine intakes should cover the metabolic requirements of the main target organs of TD: heart, peripheral and central nervous systems, muscles, and others.^5^, ^6^ The brain is particularly sensitive to any thiamine deprivation, especially during early childhood and various periods of adolescence.^7^

The prevalence of this life‐threatening disease is unevenly distributed around the world. TD is highly prevalent in low‐ and middle‐income countries (LMICs) or in humanitarian emergencies, where various limited resources cause either malnutrition or hunger with severe food insecurity.^8^ It can also occur in the context of a monotonous diet or diets that rely on low‐thiamine staple foods (e.g., polished rice), notably in breastfed infants whose mothers are thiamine deficient.^8^, ^9^, ^10^, ^11^, ^12^

Many factors have likely contributed to the reduction of this public health concern, especially in high‐income countries (HICs), such as significant advancements in large‐scale fortification of widely consumed foods (e.g., wheat flour), although thiamine fortification policies are not mandatory in a number of countries.^13^, ^14^, ^15^, ^16^, ^17^, ^18^ The advances in the diagnosis and treatment of Wernicke encephalopathy (WE) have also contributed to the reduction of its prevalence and burden.^19^ As a consequence, there are no studies that have systematically assessed TD in pediatric patients in HICs, and there is only sparse literature in the form of case reports, which contributes to the perception that pediatric thiamine deficiency (PTD) is somewhat anecdotal in HIC.^20^, ^21^, ^22^, ^23^ There are no accurate statistics available on the incidence of PTD, which is thought to be associated with a few underlying conditions.^24^

Therefore, the aim of this study is to identify cases of PTD reported in HICs during the past 20 years, including a description of the ages, causes of TD, and underlying conditions, as well as clinical manifestations of the affected patients.

## Methods

An electronic literature search was conducted on Medline (via Pubmed), Cochrane library, and Google Scholar using the cross‐referenced combination of the following MeSH terms: thiamine deficiency (TD) or disorders, thiamine metabolism, dry beriberi, wet beriberi, WE, heart failure, Shoshin, and lactatemia. Retrieved publications were screened and selected by two authors, B.R. and L.H. (and verified by third author F.G.), following the inclusion criteria: confirmed cases of TD in the pediatric population that were observed in HICs. TD was defined as positive clinical response to thiamine administration or biochemical confirmation of TD (including genetic sequencing) or specific radioimaging findings. The definition of HICs followed the World Bank classification,^25^ where low‐ and middle‐ income economies are defined as those with a gross national income per capita of $12,535 or less and high‐income economies are those with a gross national income per capita of $12,536 or more. We followed the American Academy of Pediatrics^3^ age groups definition but excluded late adolescents since most of the pediatric units do not report patients above 18 years of age. Neonate (birth to 28 days old), infancy (1 month–2 years old), childhood (2−11 years old), and adolescence (12−18 years old) were considered for inclusion.

This paper focused on the clinical aspects of PTD (as per the study authors’ judgment) and did not cover either laboratory techniques or discuss clinical–laboratory agreement.

The searches were limited to studies published between 2000 and 2020, but there were no language restrictions. The extracted variables for the included studies were: country where the study was conducted, age of the identified cases, causes of TD or underlying conditions (which were classified into categories of predisposing factors), clinical manifestations, and response to thiamine supplementation (as positive or negative, when reported). Variables were reported as numbers or percentages.

## Results

A total number of 110 articles were included, covering 389 pediatric patients under 19 years old with confirmed TD in HICs. According to the abovementioned methodology, 74% of all reports, not including genetic sequencing, assessed and had data available on serum thiamine (49 reports), whole blood thiamine diphosphate (5 reports), and erythrocyte transketolase activity coefficient (10 reports).

### Country of origin of included studies

**Figure 1:**
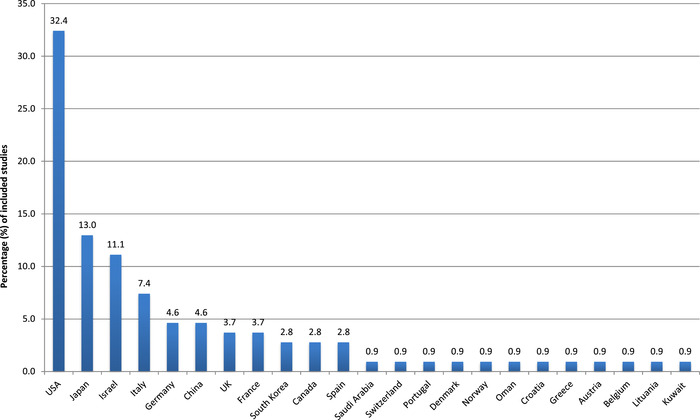
Percentage of included studies per country (case reports/series of pediatric thiamine deficiency published in 110 papers between 2000 and 2020).

The regional distribution of the included publications is displayed in Figure 1. Most of the studies originated from the United States (32.4% of all studies), followed by five countries, namely, Japan, Israel, Italy, Germany, and China. These represent the top six countries among a total of 23 countries.

### Patient age distribution

**Figure 2:**
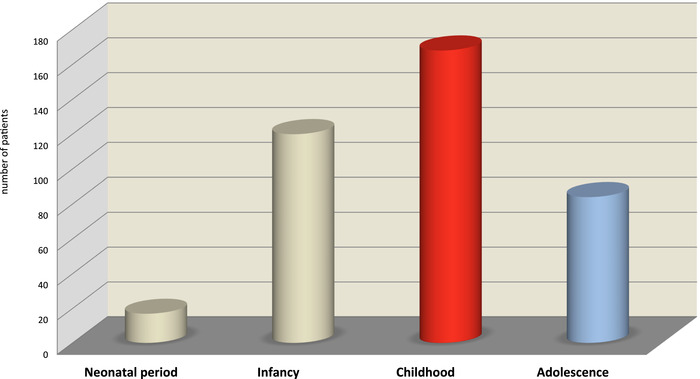
Distribution by four pediatric age groups of the overall 389 patients extracted from the 110 included studies (2000–2020).

Figure 2 shows that although infants still represent a large group, the majority of affected PTD patients in HICs are children and adolescents, as opposed to PTD observed in LMICs, where the classic infantile beriberi is well described.

### Circumstances surrounding PTD in HICs and predisposing risk factors (causes or underlying conditions)

**Figure 3:**
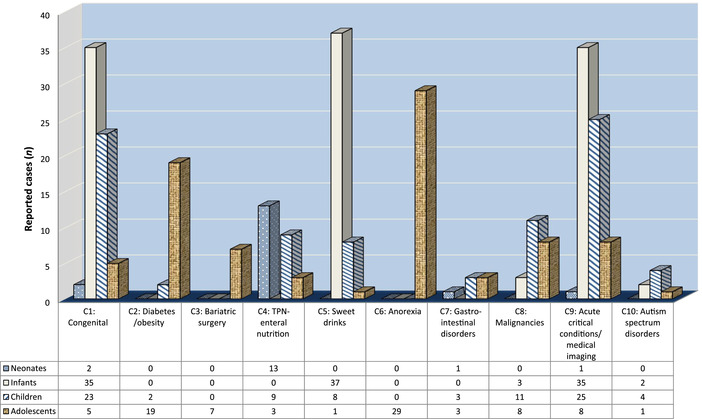
Distribution of the included cases into 10 categories of predisposing risk factors according to the four pediatric age groups.

The case reports identified (*n* = 389) can be divided into 11 main clinical categories (C0–C10) where PTDs have been described. In some conditions, multiple pathophysiological mechanisms might be involved. Table S1 (online only) presents a separate group (C0) of 91 patients described with neurological impairment as a result of earlier TD insult. Table S2 (online only) summarizes the remaining 298 included cases of PTD (C1–C10) divided by categories of predisposing risk factors (causes and underlying conditions), including both genetic thiamine disorders and acquired TD reported in HICs. Figure 3 shows the distributions of included cases into the 10 (C1–C10) categories of predisposing risk factors (causes and underlying conditions) of TD.

Below is the description of these 11 categories, classified from C0 to C10:

**C0**: PTD as a result of early infancy consumption of thiamine‐free formula, followed up in tertiary neurocognitive referral settings. This category is a large group, comprised of mostly young children followed up beyond infancy for neurological sequelae and disability following TD insults during early infancy. They present with neurological and cognitive disorders and are referred to specialized services tied to neuroimaging platforms.^20^, ^26^, ^27^, ^28^, ^29^, ^30^ This category (*n* = 91) is presented separately in Table S1 (online only).
**C1**: PTD caused by genetic inborn errors of thiamine metabolism. They account for 22% of all 298 cases and are presented in Table S2 (online only). As expected, neonates and infants represent 57% of the 65 reported cases in this category. Interestingly, children and adolescents represent 35% and 7.7%, respectively.

Using high‐throughput genomic screening, four genetic mutations and their variants have been described in 19 countries, some of them with high incidence of inborn errors of thiamine metabolism disorders that might be increased by consanguinity.^31^, ^32^ These conditions are characterized by a variable onset of the disorder and a wide range of clinical signs, including recurrent episodic encephalopathy, dystonia, convulsions, ocular abnormalities, microcephaly, anemia, thrombocythemia, and diabetes.

Three of the genetic mutations encode for thiamine transporters, and one encodes for the enzyme thiamine pyrophosphokinase 1 (TPK1), which converts thiamine into its active form:

1. Mutations in *SLC19A2*, which cause thiamine‐responsive megaloblastic anemia (TRMA), were described in eight studies (UK, Denmark, China, Portugal, Lithuania, United States, Italy, and Croatia). It is often revealed by a neonatal diabetic ketoacidosis (DKA).^31^, ^33^, ^34^, ^35^, ^36^, ^37^, ^38^, ^39^
2. Mutations in *SLC19A3*, which cause biotin/thiamine‐responsive basal ganglia disease characterized by episodes of severe Leigh‐like encephalopathy, were described in seven studies from six countries (Spain, Saudi Arabia, UK, France, Japan, and Norway).^40^, ^41^, ^42^, ^43^, ^44^, ^45^, ^46^
3. Mutations in *SLC25A19* (called Amish microcephaly) were identified in five studies (Canada, United States, Oman, Israel, and Germany).^47^, ^48^, ^49^, ^50^, ^51^ They are characterized by a very small head at birth, seizures, and episodes of lactic acidosis.
4. Mutations in *TPK1* were found in four studies (China, United States, Austria, and Germany).^32^, ^52^, ^53^, ^54^ TPK deficiency can present as episodic encephalopathy or Leigh syndrome–like early‐onset global developmental delay.

**C2**: PTD related to diabetic patients with ketoacidosis and/or obese patients (7%). In type 1 diabetes, the main concern is DKA with the occurrence of suboptimal thiamine levels even prior to insulin therapy. Then, insulin administration induces PTD. Altered mental status is a common finding of DKA, and therefore TD‐related encephalopathy could be missed.^55^, ^56^
**C3**: PTD after bariatric surgery (2%). Clinical signs of TD can occur in adolescents in the first months following surgery. The alerting symptoms are intractable vomiting or minor neurological signs preceding the onset of WE with associated hyperlactatemia.^57^, ^58^, ^59^, ^60^, ^61^
**C4**: PTD related to total parenteral nutrition (TPN) or enteral nutrition (EN) (8%) may occur at any age, even in preterm neonates or very low birth‐weight infants and infants suffering from gastrointestinal disorders or postoperative problems. Parenteral/enteral formulations might contain inadequate levels of thiamine for this age group. These patients can also present signs, such as the classic form of TD, refractory lactic acidosis, myocardial injuries, shock with multiorgan failure, and WE. Most of the time the abnormalities can be resolved with the rapid administration of thiamine supplementation.^62^, ^63^, ^64^, ^65^, ^66^, ^67^, ^68^
**C5**: PTD related to high consumption of sugar‐sweetened beverages or isotonic drinks, either in infancy or childhood/adolescence (15%).^22^, ^69^, ^70^, ^71^, ^72^, ^73^, ^74^, ^75^, ^76^, ^77^, ^78^ Most of the cases were reported in Japan. A Japanese survey revealed 33 children with regular intake of soft drinks who developed dramatic TD (confirmed by whole blood TDP).^77^ Of these, TD was clinically expressed as classical WE in four cases, one resulted in death, and 12 had long‐term neurological sequelae of TD.
**C6**: PTD related to eating disorders (anorexia) (10%).^79^, ^80^, ^81^, ^82^, ^83^, ^84^ PTD related to eating disorders consistently occurs during adolescence, based on case reports. Two large case series referring to TD‐related anorexia were retrieved.^79^, ^83^ The largest case series were in pediatric psychiatric units and the majority of cases suffered from Wernicke syndrome. The prevalence of biologically confirmed TD was estimated at 38% of the 37 patients at a specialized pediatric psychiatric unit,^79^ while this prevalence only reached 10% in a prospective study of 39 girls admitted to an outpatient day hospital for eating disorders.^83^
**C7**: PTD related to gastrointestinal disorders with reduced thiamine absorption (2%). This encompasses a wide panel of clinical conditions from intestinal resection (short bowel syndrome) to inflammatory bowel disease in children.^85^, ^86^, ^87^, ^88^, ^89^, ^90^, ^91^
**C8**: PTD related to pediatric malignancies (such as leukemia and neuroblastoma) constantly associated with hyperlactatemia and patients undergoing bone marrow transplant (7%).^92^, ^93^, ^94^, ^95^, ^96^, ^97^, ^98^, ^99^, ^100^, ^101^, ^102^, ^103^, ^104^, ^105^, ^106^, ^107^ Of note, three cases of drug‐induced TD (namely, methotrexate and metronidazole) have been included in this review.^87^, ^93^, ^101^
**C9**: PTD related to acute critical illnesses, including pediatric emergency room, surgical setting, pediatric intensive care unit (PICU), pediatric cardiology and nephrology/dialysis units, imaging facilities, and other subspecialty emergency platforms (23%). This category gathers diverse pathologies of various degrees of severity occurring in the very preterm neonatal period until late adolescence. Diverse TD expressions are described, with clinical, biological (associated hyperlactatemia), and radiologically specific or atypical signs of TD. The cases cover a broad panel of clinical manifestations: shock, pulmonary arterial hypertension, Shoshin beriberi, type B lactic acidosis (reversible by thiamine administration), altered mental status that can progress to coma, limb weakness, ocular abnormalities, and signs of truncated or the entire triad of WE.^23^, ^108^, ^109^, ^110^, ^111^, ^112^, ^113^, ^114^, ^115^, ^116^, ^117^, ^118^, ^119^, ^120^, ^121^, ^122^, ^123^, ^124^, ^125^, ^126^, ^127^
**C10**: PTD related to a few documented cases of autistic spectrum disorders associated with TD and a case of botulism associated with thiaminase production (2%).^128^, ^129^, ^130^, ^131^, ^132^

In summary, as opposed to LMICs, many cases of PTD in HICs occur in childhood and adolescence. Both age groups seem to display different underlying medical conditions leading to TD.

### Clinical manifestations of PTD

**Figure 4:**
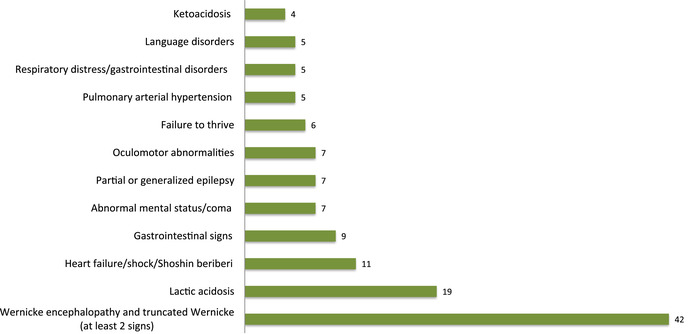
Proportion of the main clinical manifestations of pediatric thiamine deficiency described in the included studies (each case can have one or more signs).

Aside from the case series and reports of disabled patients following TD neurological insults during early infancy, as well as those with entry points of medical imaging or post mortem studies, the main clinical conditions/signs reported are displayed in Figure 4 (expressed in percentage, where each case can present with one or more of these conditions/signs).

#### Acute or insidious onset

Contrary to the mainstream medical belief that WE is limited to adults, this study highlights that acute or insidious‐onset WE is the most frequent initial sign. Indeed, 42 out the included 110 studies describe full or truncated WE, which can benefit from a cerebral imaging investigation. The clinical presentation of PTD is broad: severe or refractory underlying TD‐associated hyperlactatemia with acidosis, non‐Wernicke neurocognitive signs (sensorimotor signs or ataxia, altered consciousness, ocular signs, seizures, and dysarthria), and cardiovascular disorders (critical heart failure and shock). Special attention should be given to pulmonary arterial hypertension, remarkably reversed by thiamine supplementation. Failure to thrive is frequently reported and should not be forgotten, as it is a common source of medical referral to pediatricians.

#### Long‐term sequelae

These mostly include encepalopathy, epilepsy, and language disorders. Small outbreaks of classic beriberi in breastfed infants from thiamine‐deficient mothers in the French islands of Mayotte,^133^ and the unfortunate incident of thiamine‐deficient soy‐based infant formula in Israel^30^ that jeopardized the neurocognitive outcomes of young patients in the long term, remind the medical community that the classic forms of beriberi in their most detrimental outcomes can still occur in HICs.

It should be also noted that in Israel a number of infants who received the same thiamine‐free, soy‐based formula but who were asymptomatic still presented with later gross‐ and fine‐motor function alterations and learning difficulties.^27^, ^134^

PTD caused by genetic inborn errors of thiamine metabolism cases (C1) can occur in HICs and especially in migrant and/or consanguineous families.^31^, ^32^

### Response to thiamine supplementation

We chose the following criteria to report whether the patients responded to thiamine supplementation, as it is not always been clearly documented: the authors’ judgment, clinical or radiological signs of improvement, lactatemia dynamics, anemia response, and change in the required dose of insulin or vasopressor drugs in response to thiamine (Tables S1 and S2, online only). A positive clinical response to thiamine administration was identified in 90 of the 110 reports in this review (82%).

## Discussion

Despite the heterogeneity in the methodology of reports, they have been grouped into 10 categories (C1–C10) of main underlying conditions potentially leading to PTD. Some case reports could fit in two or more categories, but in order to avoid confusion, they were classified in only one category. These underlying conditions may have one or multiple pathophysiological mechanisms that can lead to TD: (1) low vitamin intake (i.e., an absolute insufficiency in exogenous thiamine intake through foods, drinks, mother's deficient milk, EN or TPN, and/or poor intestinal absorption via impaired intestinal thiamine absorption regardless of the cause); (2) thiamine to caloric ratio imbalance (i.e., an inadequate thiamine intake in comparison with the amount of energy consumed or a mismatch with endogenous hypermetabolic requirements, such in fever, severe illnesses, and infections); (3) cellular machinery dysfunction (i.e., a defect in thiamine transport or an impaired cellular utilization due to a reduced level of thiamine pyrophosphate, the active intracellular form of thiamine); (4) excessive losses (i.e., increased renal or digestive loss, as in renal or peritoneal dialysis, or use of diuretics, or by recurrent diarrhea or vomiting, or postsurgery‐induced hyperemesis); and (5) metabolites or drug interactions with thiamine metabolism (i.e., oxythiamine, thiaminase, or methotrexate and metronidazole).

The fact that one third of the publications are from the United States can be explained by many factors and does not necessarily represent a higher prevalence of PTD in this country. First of all, although there were no language restrictions, most included publications were written in English. The impact of the modern lifestyle on pediatric noncommunicable diseases (NCDs), such as severe obesity and type 2 diabetes, but also diet and drinking habits (of sugar‐sweetened drinks) in young children and adolescents, is particularly noted in the United States. There is likely a reporting bias as the pediatric workforce in the United States is one of the highest in the world; as such, access to pediatric research is common, and therefore the reporting of PTD is likely to be higher.

### PTD age‐related specific vulnerability in HICs

In neonates, the category C1 shows a predominant proportion of genetic thiamine disorders with a fixed incidence related to the four most common mutations. They are usually managed by neonatologists and geneticists. The current state of wide globalization is associated with increasing movement of populations; and cases affected by these hereditary disorders, enhanced by consanguinity, might appear in locations where it has not been described previously.^31^, ^32^ These rare conditions need urgent management as some of them respond to thiamine, such as TRMA. The advances in modern neonatal care have also increased the survival rate of extremely low birth weight preterm babies who are also at high risk of developing TD through a combination of several complex factors, including very limited thiamine stores and organ immaturity with rapid growth. Therefore, they exhibit a significant dependence on the dual‐nutrient “carbohydrate‐thiamine” supply,^135^ and inadequate intake or mismatch (not enough thiamine supply compared with carbohydrate supply) with TPN or EN can lead to severe TD.^62^, ^67^

Children and adolescents represent 65% of all reported cases in this study. In infancy, three categories are predominantly represented (C1, C5, and C9). In childhood, four categories clearly emerged as prominent causes of TD. Category C0, corresponding to the follow‐up of patients who survived TD insults during infancy, is the predominant category, followed by critically ill children in several emergency and subspeciality settings (C9), children on TPN or EN (C4), and then soft drinks habits, as in category C5. The adolescence period is dominated by anorexia (C6) and the combined categories C2 and C3, both related to NCDs.

Thus, the age of the population appears to be the most important determinant that has shaped the current pattern of PTD in HICs, out of the classic TD forms that previously prevailed and still remain in LMICs. The age groups mostly affected by PTD display a shift from early infancy (frequently observed in LMICs) toward childhood and, to a lesser extent, adolescence (in HICs). Another potential explanation for this difference is the higher diagnostic capacity of PTD in HICs, representing a potential bias. It is possible that if those diagnostic capacities were available/accessible in LMICs, more children and adolescents could be diagnosed with PTD.

### Impact of the modern lifestyle in HICs and the growing burden of NCDs on thiamine metabolism in pediatrics

#### Excessive consumption of soft drinks in the pediatric population

This literature review raises two important points about two periods of the pediatric lifespan.

On the one hand, there is the description of a rapid onset of acute TD in infants who have an excessive consumption of soft drinks, as shown in category C5. Okumura *et al*. report a series of concerning and ongoing PTD cases in Japanese infants and young children (7–30 months old) as a result of regular consumption of an average of 1000 mL/day of iso‐/hypotonic soft drinks in nonnurturing environments.^77^ Reports include cases of heart failure, pulmonary arterial hypertension, and WE consistently reversed with additional thiamine, and neurological sequelae and death in some.

On the other hand, there is the slow build‐up, insidious TD resulting from the consumption of sweetened sugar drinks in childhood and adolescence. Continuing from childhood habits, the obesogenic effect of sugar‐sweetened beverages is extended into adolescent dietary patterns associated with the consumption of carbonated soft drinks and calorie‐dense snack foods.^136^, ^137^, ^138^, ^139^, ^140^, ^141^ Without thiamine supplementation, this can lead to PTD. The availability of varied and balanced diets, such as those provided by school meal programs, may mitigate the expression of TD, rarely overtly observed in this population. Adolescent binge drinking, often associated with excessive carbohydrate consumption, puts this group at risk of TD and particularly of WE.^78^, ^142^

#### Diabetes

During the last two decades, the burden of type 2 diabetes and “diabesity” (the association of obesity with type 2 diabetes) has dramatically increased.^143^, ^144^, ^145^, ^146^ Pediatric type 1 diabetes incidence is also increasing, and almost half of them are in European or North American countries.^147^

DKA is the most common life‐threatening complication of pediatric type 1 diabetes and is occasionally associated with cerebral damage,^148^, ^149^ and it has been described in all age ranges, including in neonatal diabetes associated with TRMA syndrome.^31^, ^34^, ^148^ Ketoacidosis is a common reason for the discovery of diabetes in children and results in a subclinical cerebral edema that progresses (even in the first hours of insulin therapy) to encephalopathy.^55^, ^56^, ^149^ The DKA‐related cerebral edema associated with hyperlactatemia is still not completely understood in some respects.^150^, ^151^ In addition to osmotherapy,^148^, ^152^ recent data suggest that thiamine might have a potential adjuvant role in the global management of DKA^55^, ^148^, ^150^, ^153^, ^154^ A positive correlation is also observed between diastolic myocardial dysfunction and serum thiamine levels in children with DKA.^155^ Thus, converging evidence suggests that thiamine may exert a beneficial effect in the management of DKA in children and even those with TRMA.^34^, ^56^, ^155^ Unlike in adults, no studies have addressed the potential effect of thiamine to prevent specific complications in the course of chronic pediatric diabetes.^156^, ^157^, ^158^

#### Severe obesity and bariatric surgery

The prevalence of overweight and obesity in children is plateauing after many years of sharp increase in HICs.^159^, ^160^ However, morbid obesity still continues to grow silently in an obesogenic environment, most notably in 12‐ to 19‐year‐olds.^159^, ^161^, ^162^ Despite multidisciplinary programs to manage pediatric obesity, bariatric surgery is one of the primary management methods. Operations are costly and constraining, using either restrictive or gastrointestinal bypass procedures now extensively performed by well‐trained surgeons in HICs, even in pediatric populations.^163^

It is a common belief that TD after bariatric surgery is a frequent complication that occurs insidiously within 6 months after surgery in pediatrics, as in adults. No recent large studies or cohorts have yet established the true prevalence of TD in pediatric bariatric surgery patients.^164^ Conversely, the literature abounds with scattered and somewhat informative case reports on TD in obese children after pediatric surgery,^58^, ^164^ requiring mulidisciplinary teams. Postbariatric TD usually presents as episodes of repetitive, uncontrolled diarrhea or vomiting, associated with minor neuropathic symptoms or atypical ocular signs, or full blown WE.^165^ Our review suggests that most cases of WE occur in adolescents, indicating a profound depletion of the body's thiamine reserves over time.

Deficiencies of micronutrients, including vitamins D and B12, folate, iron, and thiamine, are also reported prior to bariatric surgery, likely due to attempts to lose weight tied to self‐induced vomiting or an unbalanced diet.^166^

#### Eating disorders and anorexia

Anorexia in adolescence (C6) occupies a predominant proportion of cases in this review. In most HICs, these patients are managed by specialized multidisciplinary eating disorder units. Eating disorders, including anorexia, have also become a significant public health issue among adolescents in the industrialized world^167^, ^168^, ^169^ and middle‐income countries (MICs)^170^ They affect mostly girls, with an initial onset between 10 and 20 years of age and a peak age of onset in early to mid‐adolescence, according to studies in six European countries.^171^ Two studies have described a variable frequency of anorexia‐related TD of between 10% and 32%.^79^, ^83^ Anorexia is a complex neuropsychiatric disorder with multifactorial components (sociocultural, epigenetic, and genetic).^172^ Our review shows that anorexia is associated with various phenotypes of TD, WE being the culminating neurological presentation associated with other reported minor neuropsychiatric symptoms.^79^, ^80^, ^81^, ^82^, ^84^ The multiple mechanisms leading to TD in anorexia include malnutrition associated with poor intake, as well as laxative use and self‐induced vomiting in patients with purging behaviors. A hypothesis for the occurrence of WE could be that pronounced depletion of thiamine stores and inadequate intake of thiamine affects the brain that is still undergoing maturation in adolescence^7^, ^10^ An underlying genetic error of thiamine metabolism has been described in some patients, making them more susceptible to any thiamine‐deficient diet.^19^, ^172^, ^173^ Refeeding requires special attention in these cases.^174^

### To what extent can higher diagnostic capacities in HICs influence the detection of clinical expressions of PTD compared with resource‐limited settings?

**Figure 5:**
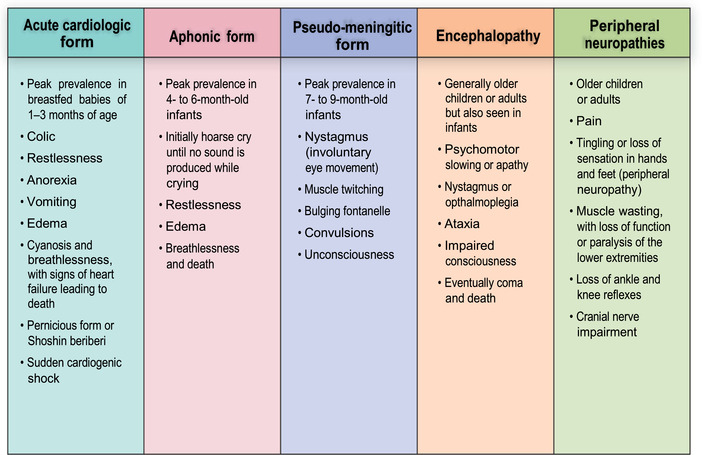
Clinical spectrum of classic thiamine deficiency. Reproduced from Ref. 9.

The classic TD clinical spectrum is summarized in Figure 5. These clinical presentations are discussed in recent publications.^9^, ^175^, ^176^

#### Classic infantile beriberi

Classic infantile beriberi in early months of infancy caused by feeding by thiamine‐deficient mothers is very rarely observed in HICs. Only two sets of cases are reported in this review. The first occurred on an epidemic scale in families living in disadvantaged socioeconomic conditions on the French island of Mayotte. Some sporadic cases have also been observed in the island of Reunion.^109^, ^133^ The second case series is related to the unfortunate case of infants fed thiamine‐deficient soy milk formula, which occurred on a large scale in Israel in 2003^30^, ^122^ and had a major long‐term impact, as discussed below.

In this study, there was no report of the nonspecific signs associated with infant TD, such as loud piercing cries, colic, refusal to breastfeed, vomiting, and agitation.^177^, ^178^ Nevertheless, our current review may suffer from underreporting of this early form of TD because most studies originate from specialized settings and not from primary care pediatrics. Few available studies have addressed the issue of the micropockets of poverty that are consistently present in the large megacities of rich countries. This context specifically relates to the hidden and high‐risk young population susceptible to overlooked multiple nutritional deficiencies, in addition to infectious and/or respiratory diseases within the general framework of pathology affecting minority families, poor migrants, and even homeless breastfeeding women, who can be marginalized from access to primary and specialist care.^179^, ^180^, ^181^, ^182^

#### Cardiovascular beriberi

Cardiovascular beriberi consists of the onset of heart failure, which can occur suddenly. It is described as hyperlactatemia associated with a high‐output cardiac failure, predominantly in the right or both cardiac chambers, and in its edematous form with a pitting edema in the lower limbs. The outcome can be exacerbated by loop diuretics without prompt thiamine administration.^66^, ^113^, ^114^, ^125^

One of the major findings was the occurrence of acute respiratory distress in tachycardic infants, expressed as a pulmonary arterial hypertension with hyperlactatemia, which is remarkably reversed by thiamine treatment, as noted earlier.^72^, ^74^, ^111^, ^125^, ^127^, ^131^

The popularization of pediatric bedside cardiac Doppler ultrasound in infants and the readily available assay of lactate point‐of‐care testing have allowed such a refined diagnostic and therapeutic achievement, which is becoming routine practice in HICs.^183^ However, case reports of TD related to pulmonary arterial hypertension were emerging not long ago in MICs, where ultrasound cardiac Doppler and trained local staff are becoming more available.^184^, ^185^, ^186^ This highlights that LMICs and HICs differ more from diagnostic capacities than clinical presentations.

#### Neurological signs of PTD

Early minor manifestations of TD neuropathy are characterized in this review by symmetrical sensory‐motor impairments (such as muscle pain and weakness),^84^ dysarthria, dysfunction or paralysis of the lower extremities, progressive (gait) ataxia with or without inaugural signs of encephalopathy, and decreased deep tendon reflex function of the extremities, affecting mainly the lower limbs. This can progressively lead to altered consciousness.^80^, ^82^, ^84^, ^108^, ^122^

The diagnosis of unexplained severe neurological signs of encephalopathy can benefit from modern neuroimaging and/or response to thiamine administration or both. In this review, there is no report of pseudo‐meningitic forms that usually occur around 6−12 months of age.^8^, ^187^ WE was the most frequent manifestation of TD in our review (Fig. 4), which can occur at any age,^57^, ^89^ but a significant subset of the reports in this review are during adolescence, as seen in categories C7–C9, as opposed to infancy in LMICs.^188^, ^189^ Its onset can have a rapid progression, with or without ataxia, and can be associated with other minor clinical manifestations, including muscular fasciculation or weakness, atypical ocular symptoms, nystagmus, ophthalmoplegia, seizures, and altered mental status until coma.^59^, ^61^, ^65^, ^122^, ^173^ The main causative pathological conditions in our review were from anorexia, morbid obesity, and bariatric surgery; all of them are caused by an insufficient supply of thiamine tied to the lack of risk awareness.^62^, ^90^, ^105^

In pediatric medical imaging platforms, as found in tertiary hospitals in HICs, the imaging modalities are useful tools for assessment and making a rapid as well as decisive diagnosis of PTD. Magnetic resonance imaging (MRI) is more available and feasible in HICs as a screening modality because of its greater diagnostic potential over CT. In adult studies, the MRI sensitivity is 53%, but its specificity is 93%, with a positive predictive value of 89%.^190^ However, it is now widely used in pediatrics in HICs.^191^ Unlike in adults, the pediatric brain MRI of Wernicke‐like syndrome can display peculiar lesions in the frontal lobe and basal ganglia, notably the striatum and putamen, all of these regions being sensitive to thiamine deprivation. Atypical lesions can also be located in the cerebellum. An evaluation of the cortex serves as an indicator of prognosis.^191^, ^192^, ^193^ However, adults and children affected by TD show the same symmetrical high‐intensity signal on T2 weighting in mammillary bodies and periaqueductal and thalamic areas, sites of neuronal damage, and disruption to myelination confirmed by cerebral spectrum MRI.

Additional diagnostic tools can also be useful in identifying and characterizing TD. Cranial transfontanelar ultrasound in infants could be an alternative to MRI, as it can be performed at the bedside (point of care), revealing hyperechoic lesions of the basal ganglia.^188^ Other techniques, such as the use of contrast enhancement, fluid‐attenuated inversion recovery (FLAIR) images, and diffusion‐weighted imaging,^194^ as well as MR spectroscopy (displaying high lactate peaks),^195^ have additional diagnostic value.^195^ In addition, MRI can be used to monitor *in situ* recovery of neurological damage during thiamine treatment.^191^, ^196^

The MRI findings in HICs and LMICs are similar, with basal ganglia involvement (especially putamen and caudate nucleus^20^, ^77^, ^189^, ^191^), and the two differ largely by the accessibility of such neuroimaging tools. Some differences found between LMIC and HIC cohorts^189^ regarding less involvement of other cerebral regions (e.g., mammillary bodies and periaqueductal gray matter) might be explained by multiple factors. Mammillary bodies and periaqueductal area abnormalities seem to be more frequent in children and adolescents than in young infants.^23^, ^60^, ^61^, ^68^, ^82^ Similarly, nutrition, environment, and genetic diseases (such as Leigh syndrome) potentially overlapping with the TD spectrum might also contribute to these differences.^188^, ^189^, ^197^ MRI protocols, such as contrast enhancement, might increase the sensitivity for mammillary bodies detection and has not been used systematically in some studies.^20^, ^23^, ^198^, ^199^, ^200^, ^201^

Neurological presentations, such as WE in adolescents, seem more frequent in HICs than in LMICs, but this may be biased as neuroimaging is the standard of care in HICs and has contributed significantly to its diagnosis.

Long‐term neurological sequelae (C1) was the most commonly reported of the categories in children. It includes outpatient care in pediatric neurology departments, or follow‐up in pediatric neurological services or neuroimaging department.^26^, ^27^ They are investigated in the neurolinguistic and psychological settings for their alteration in the syntactic and lexical modalities of language acquisition.^28^ Most overt symptomatic cases who survived later developed complications in the form of epilepsy or lack of acquisition of developmental milestones.^29^ Some children also presented permanent auditory dysfunction.^134^

A number of infants who received this thiamine‐deficient soy‐based formula in Israel, but who were initially asymptomatic still presented years later with gross‐ and fine‐motor function alterations and learning difficulties.^9^, ^28^ This reflects how important thiamine is for children's overall neurodevelopment, and it highlights the importance of early diagnosis of TD and subclinical TD during this critical period in infancy of neurodevelopment that involves the acquisition of cognitive skills. *In utero* exposure to TD may also lead to fetal brain dysfunction, as observed in fetal alcohol syndrome.^202^

This nutritional incident in Israel has probably contributed to reducing the extent of TD in infants in HICs because many national regulatory agencies in every industrialized country, the major international pediatric societies, and the infant formula industry have complied with the current minimum standards for infant formula,^203^ in addition to the WHO promotion of breastfeeding in the first 6 months of life. However, such poor outcomes may be the fate of children from very underprivileged or homeless families who gradually develop TD, which can be expressed months or years later as neurocognitive disabilities.^182^

### High‐level medical management of pediatric complex illnesses can be associated with PTD in HICs

Most of the cases from this review occurred in high‐level tertiary centers, as opposed to classic beriberi in LMICs that tends to occur in primary settings. This is in line with the Lallas and Desai review^173^ that describes WE in children and that points out three main scenarios, such as bariatric surgery, malignancies, and intensive care management. Our study reveals similar findings with more recent and detailed categories.

Owing to the increasing prevalence of morbid obesity and the recent advances in bariatric surgery procedures,^204^ more and more adolescents follow this option, as represented by the C3 category. Bariatric surgery is a highly specialized, requiring multidisciplinary teams, including nutritionists, among other specialists. The reduction of both thiamine absorption following surgery and thiamine intake in the first months can lead to severe TD, including WE.^57^, ^58^, ^59^, ^60^, ^61^

Categories C8 and C9 include diverse critical conditions that increase the risk of developing TD. Many highly specialized pediatric services were involved in these categories: oncology units, pediatric emergency rooms, PICUs or neonatal intensive care units, specialized surgical services (abdominal, cardiac, and transplantation), gastroenterology, and imaging and dialysis units. This is also reflected by the large number of different critical conditions, including cases of coma, shock with hyperlactatemia, heart failure, pulmonary arterial hypertension detected by color Doppler sonography, postsurgical nutritional complications, nephrological cases, and neurological cases with minor sensorimotor signs or atypical or full WE.^23^, ^91^, ^110^, ^111^, ^116^, ^119^, ^123^, ^125^, ^126^, ^131^

In these categories, multiple pathogenic mechanisms leading to TD are often involved. This can be observed in postsurgical cases of heart operation or organ transplantation.^119^ The predominant cause is a mismatch between an increased metabolic demand and an inadequate thiamine availability. This can be caused by the coexistence of underlying malabsorption or excessive digestive or renal loss of thiamine, and/or insufficient removal of oxythiamine (a uremic toxin inhibiting transketolase), as in end‐stage renal disease.^205^, ^206^, ^207^ As such, an unusual cluster of TD cases has been reported to be mostly tied to advanced techniques in dialysis or hemofiltration.^114^, ^121^, ^126^ Such a subcategory of PTD risk linked with end‐stage renal diseases has not been fully delineated by Lallas and Desai,^173^ while there has been significant and incremental advances in renal replacement‐dialysis modalities (peritoneal dialysis, intermittent hemodialysis, and continuous and hybrid therapies).^208^

C4 is represented by a set of reports of neonates and infants/children on TPN or EN with poor thiamine intake or increased intestinal losses in which TD appears after insufficient thiamine supply in therapeutic feeding.^62^, ^67^

Critically ill patients, often malnourished and/or on prolonged TPN, have complex metabolisms that can be further compromised by receiving polychemotherapy that can interact with thiamine metabolism: diuretics, proton pump inhibitor drugs, metronidazole and some other antibiotics, tacrolimus, cyclosporin,^209^, ^210^ 5‐fluorouracil, and methotrexate,^93^, ^101^ among others. This is represented to some extent in our C8 category, especially in perioperative bone marrow transplantations; however, drugs that potentially interact with thiamine metabolism are not uncommon in other categories. Transient reduction of micronutrients has been described in systemic inflammation,^211^, ^212^, ^213^ and Peixoto de Lima *et al*. described an independent correlation with C‐reactive protein (CRP) levels and TD.^214^ In this review, very few reports mention CRP monitoring,^70^, ^85^, ^86^, ^87^, ^117^, ^127^ and none monitored CRP and thiamine trends simultaneously, apart from Peixoto de Lima *et al*. The not insignificant consequences of transient TD on children's developing brains are difficult to document in daily clinical practice as no clinician would take the risk of delaying such treatment (due to the potential long‐term sequelae that have been described even in initially asymptomatic thiamine‐deficient infants, as documented in the C0 category of this study).

While the major cause of classic diet thiamine deprivation is no longer relevant in 2020 in HICs, this study has revealed a small but noticeable number of PTD cases arising from critical illnesses or chronic conditions that are managed in highly specialized pediatric services. All of these patients suffer various risks of PTD.

### Strengths and limitations

#### Strengths

This up‐to‐date review is a condensed and unique study that comprehensively addresses complex PTD issues, including age, clinical presentation, and predisposing risk factors, observed in HICs.

#### Limitations

First, as this is not a systematic review, the number of retrieved articles cannot reflect the magnitude or prevalence of overall PTD in HICs. The number of countries included in this review accounts for only 28% of the total HICs, according to the World Bank.^25^ We also excluded opinion pieces and commentaries, which do not constitute original research.

Second, the included studies are only case studies and series, which are not evidence of the best quality (i.e., high‐quality, randomized clinical trials or systematic reviews). Nevertheless, they should not be excluded from scientific consideration. These instructive case series or reports deal with facts and are based on observational data, so they remain illustrative of the real world of medical practice in PTD issues.

Third, the methodology of using a compilation of several scattered cases led to a high level of clinical heterogeneity. Nevertheless, in light of the likely low prevalence of the phenomenon, this method proves to be the only pragmatic approach to account for it. They result in concordant PTD observations encountered all over the world within the limited circle of HICs.

## Conclusion

This study provides an updated and holistic overview of PTD in HICs over the last two decades and highlights two paradigm changes. First, there is a clear shift toward older children as opposed to young infants, as seen in LMICs. Second, multiple underlying conditions, either individually or concomitantly, can lead to PTD in HICs, such as anorexia, critical illnesses, malignancies, and renal replacement therapies. The growing burden of NCDs, such as morbid obesity, is beginning to have an impact on PTD. In addition, this study supports the idea that the nervous system, notably the growing brain, is a crucial target of thiamine deprivation among the pediatric population. WE is the predominant manifestation of PTD, and its early signs need to be known in order to avoid long‐term sequelae. Neuroimaging with MRI is a common diagnostic tool, with characteristic TD findings that allow clinicians to monitor treatment response as well. PTD is mostly described in high‐level tertiary centers, where medical awareness needs to be high as PTD can also be associated with complex medical management. The number of children with neurocognitive disabilities, followed up after an overt clinical or subclinical TD insult that occurred during early infancy, should call for special attention toward the most vulnerable populations in HICs, including pregnant migrant women and young infants living in precarious conditions in HICs.

## Author contributions

B.R. and L.H. were involved in the conception and drafting of the manuscript, performed the literature searches, and extracted the data. All authors were involved in the design (including table and figures) and revision of the manuscript, and the approval of its final version.

## Competing interests

The authors declare no competing interests.

## Supporting information


**Table S1**. TD in early infancy followed up in tertiary neurocognitive referral centers.

**Table S2**. Overall description of cases of pediatric thiamine deficiency divided by categories of predisposing risk factors.
