# Supplementary material for: Pediatric thiamine deficiency disorders in high‐income countries between 2000 and 2020: a clinical reappraisal
Source: Ann N Y Acad Sci. 2021 Jul 26;1498(1):57–76. doi: 10.1111/nyas.14669 (PMC9290709; doi:10.1111/nyas.14669)
Supplement: Supplementary file 1 — Table S1. TD in early infancy followed up in tertiary neurocognitive referral centers. [file NYAS-1498-57-s002.docx]

Supplementary Table 1 – Thiamine deficiency in early infancy followed-up in tertiary neurocognitive referral centers

| **Study author, year, country** | **Age group, n** | **Context** | **Evolving clinical signs** | **Auxiliary**  **tests** | **Response to thiamine** |
| --- | --- | --- | --- | --- | --- |
| **C0 - TD in early infancy followed-up in tertiary neurocognitive referral centers** | | | | | |
| Fattal-  Valevski,  2005,  Israel^30^ | Inf  8 | TD outbreak in infants due to defective soy-based formula,  outcome of subset of patients from TD outbreak in Israel, in 2003 | Vomiting, diarrhea, failure to thrive, lethargy, restlessness, developmental delay, nystagmus, ophthalmoplegia, intussusception, hypotonia, apnea, hyperlactatemia (serum, CSF). | ETK-AC  Lactate (serum, CSF)  Brain computed tomography, MRI | +  if timely  supplied |
| Kornreich,  2005,  Israel^20^ | Child  6 | MRI findings in PTD following TD outbreak in Israel, in 2003, secondary to defective soy-based formula | Dysphagia, d evelopmental d elay, ataxia, hyperlactatemia (serum, CSF).  WE imaging focusing on:   1. the areas tectum, brain stem, periaqueductal gray matter, mammillary bodies, thalami hypothalamus, caudate, putamen, frontal cortex, volume loss 2. high signals on T2, FLAIR   3) local changes: cytotoxic or vasogenic edema, brain volume loss (white matter), hemorrhage and necrosis in repeated MRI. | Lactate (serum, CSF)  Brain MRI, MRS  FLAIR  gadolinium | NA |
| Fattal-  Valevski,  2009,  Israel^28^ | Child  20 | Delayed language development due to infantile TD: cohort of several infants with encephalopathy from TD outbreak in Israel, in 2003 | Initial assessment : lethargy, prolonged vomiting, seizures, coma.with upbeat nystagmus, ophthalmoplegia.  Follow-up: WE, with delay in receptive language, expressive language and motor capacity. | Preschool linguistic scale  milestones acquisition  EEG | NA |
| Fattal-  Valevski,  2009,  Israel^29^ | Child  7 | [Epilepsy in children with TD](https://hdas.nice.org.uk/strategy/925090/3/PubMed/19571254),neurocognitive follow-up of the cohort from TD outbreak in Israel, in 2003 | All: either tonic, myoclonic, or focal seizures, mental retardation, motor disabilities, brainstem dysfunction  various forms (partial, hypsarrhythmia, myoclonic) of recurrent or refractory seizure on EEG. | EEG  neuromental specific testing | NA |
| Mimouni-Bloch,  2014,  Israel^26^ | Child  11 | TD in infancy: long-term follow-up of cohort from TD outbreak in Israel, in 2003 | Acute encephalopathy with bulbar and ocular signs, neuropathy, associated with cardiomyopathy and atrioventricular block.  In the long-term: vegetative state, death, mental retardation, motor dysfunction, epilepsy, kyphoscoliosis, atrioventricular block. | EEG  motor and neurological tests | NA |
| Harel,  2007,  Israel^27^ | Child  39 | Effect of subclinical infantile TD on motor function in children from TD outbreak in Israel, in 2003 | Long‐term sequelae of PTD in 39 preschoolers children versus 30 age‐matched healthy non exposed to TD.  Motor and graphomotor development impairments, some with severe motor dysfunction | Child Motor Battery and the Zuk Assessment | NA |

**Legend:**

For age range: Inf: infant; Child: childhood.

For response to thiamine: NA: not available; NC: not clear; **+**: positive

CSF: cerebrospinal fluid

EEG: electroencephalogram

ETK-AC: erythrocyte transketolase activity coefficient

FLAIR: fluid-attenuated inversion recovery

MRI: magnetic resonance imaging

MRS: magnetic resonance spectroscopy

TD: thiamine deficiency

PTD: pediatric thiamine deficiency

WE: wernicke encephalopathy
