# Supplementary material for: Pediatric thiamine deficiency disorders in high‐income countries between 2000 and 2020: a clinical reappraisal
Source: Ann N Y Acad Sci. 2021 Jul 26;1498(1):57–76. doi: 10.1111/nyas.14669 (PMC9290709; doi:10.1111/nyas.14669)
Supplement: Supplementary file 2 — Table S2. Overall description of cases of pediatric thiamine deficiency divided by categories of predisposing risk factors. [file NYAS-1498-57-s001.docx]

Supplementary Table 2 – Overall description of cases of PTD divided by categories of predisposing risk factors

| **Study author, year,**  **country** | **Age group, n** | **Context** | **List of evolving clinical signs** | **Auxiliary**  **tests** | **Response to thiamine** |
| --- | --- | --- | --- | --- | --- |
| **C1 - Inborn errors of thiamine metabolism** | | | | | |
| Thiamine-responsive megaloblastic anemia (**TRMA** or Rogers syndrome) mutation of **SLC19A2**  *(TRMA)OMIM 249270* | | | | | |
| Lagarde**,** 2004  USA^33^ | Inf  1 | N ovel gene mutation in African american | sensorineural hearing loss, restricted peripheral vision, diabetes requiring insulin | genetic sequencing | NA |
| Olsen, 2007 Denmark^31^ | Inf  3 | D [iabetes in](https://onlinelibrary.wiley.com/doi/10.1111/j.1399-5448.2007.00251.x)  [childhood](https://onlinelibrary.wiley.com/doi/10.1111/j.1399-5448.2007.00251.x)  consanguinity in Pakistani origin | megaloblastic anaemia, hearing loss,  non‐autoimmune d iabetes | genetic sequencing | NA |
| [Shaw‐](https://onlinelibrary.wiley.com/action/doSearch?ContribAuthorStored=Shaw-Smith%2C+Charles)Smith,  2012  UK^34^ | Inf  5 | N eonatal diabetes mellitus. homozygous mutations in *SLC19A2* | stroke, focal or generalized epilepsy,  visual impairment and cardiac abnormalities, neonatal diabetes | genetic sequencing | NC |
| Mozzillo, 2013  Italy^35^ | Child  2 | N ovel SLC19A2 variant with heterozygous SLC19A2 mutation | deafness, retinitis pigmentosa,  optic nerve atrophy, macrocytic anemia and type 1 diabetes.  resolution of the anemia and reduction of insulin requirement with B1 | genetic sequencing | + |
| Tahir**,**  2015  Portugal^36^ | N  1 | SLC19A2 mutation from non-consanguineous Portuguese parents | optic nerve atrophy, deafness with abnormal hearing evoked potential, short stature, anemia, aminoaciduria, diabetic ketoacidosis. | genetic sequencing | + |
| Mikstiene,  2015  Lithuania^37^ | Child  1 | SLC19A2 gene mutation with no consanguinity | impaired vision, photophobia, bilateral hearing loss, refractory anemia, diabetes requiring in insulin, | genetic sequencing | + |
| Li,  2019  China^38^ | Child  1 | C erebral infarction with mutations in the *SLC19A2* gene | diabetic ketoacidosis, anemia, deafness. leukopenia, thrombocytopenia, horizontal nystagmus hepatomegaly, short stature | Brain CT, MRI  genetic sequencing | + |
| Uroic,  2020  Croatia^39^ | Inf  2 | Patients with a SLC19A2 variant responsive to thiamine | 1^st^ patient: anemia at 4 months of age and type 1 diabetes  2^nd^ patient: anemia at 3 weeks of age | genetic sequencing | + |
| Biotin–thiamine-responsive basal ganglia disease **(BTBGD**) caused by a mutation in the **SLC19A3** gene  *(BTBGD) OMIM 607483* | | | | | |
| Debs,  2010  France^40^ | Child: 1  Ado: 1 | Encephalopathy due to SLC19A3 mutations.  Portuguese origin | both patients: swallowing impairment, dysarthria, partial motor dysfunction and inability to walk, ataxia, generalized dystonia, seizures, confusion; patient 1 expressed loss of speech and pyramidal signs while patient 2 presented with gaze nystagmus | CSF lactate  Brain CT, MRI  genetic sequencing | +  biotin |
| Yamada  2010  Japan^41^ | Inf  4 | Clinical and MRI findings in consanguineous patients with mutated SC19A3 | truncated WE with epilepsy, ataxia, nystagmus, ophthalmoplegia, and development delay | Brain MRI  genetic sequencing | +  biotin |
| Pérez-Dueñas,  2013  Spain^42^ | N  1 | R[eversible neonatal lactic acidosis with](https://pediatrics.aappublications.org/content/131/5/e1670) SLC19A3 mutation | encephalopathy, irritability, crisis of opisthotonus; B1 and biotin improved biochemical and neurological signs however the patient presented with sequelae (necrosis and volume loss in few brain areas) | lactates  acid organic  Brain MRI | +  biotin |
| Fassone,  2013  UK^43^ | Ado  1 | Treatable Leigh-like encephalopathy with *SLC19A3* mutation. | onset of ptosis, ophthalmoplegia; then ensued a subacute encephalopathy, differential diagnosis of Leigh syndrome was considered | Brain MRI  genetic sequencing | +  biotin |
| Alfadhel,  2013  Saudi Arabia^46^ | Inf 8  Child 9  Ado 1 | Biotin-thiamine-responsive basal ganglia disease: the clinical, radiological and molecular findings of  18 new cases:  notion of consanguinity | subacute encephalopathy, ataxia (n=18), seizures (n=13), dystonia (n=12), dysarthria (n=9), quadriparesis hyperreflexia (n=9);  on follow-up, 4 died, 2 had spastic quadriplegia, 6 had normal outcome and the rest had speech and motor dysfunctions;  B1 with biotin improved clinical signs in survivors | genetic  sequencing | + |
| Aljabri,  2016  Saudi Arabia^44^ | Child  1 | Thiamine responsive [in](https://journals.lww.com/md-journal/Fulltext/2016/10040/A_case_report_of_biotin_thiamine_responsive_basal.17.aspx)  a consanguineous child with SLC19A3 mutation | sudden ataxia, dystonia, hypertonia and hyperreflexia | genetic sequencing  Brain MRI | +  biotin |
| Flønes,  2016  Norway^45^ | Inf  3 | Encephalopathy: SLC19A3 mutation in non-consanguineous family | progressively suffered from hemiparesis, short distances walk and frequent falls; then developed encephalopathy consisting of dystonia, epilepsy, psychomotor regression, no speech, dysarthria, severe dysphagia | genetic test  Brain MRI  T2-FLAIR,  18 FDG -PET | +  biotin |
| Amish lethal microcephaly mutation of **SCL25A19** - phenotype obvious at birth with ketoglutaric aciduria  *(lethal microcephaly) OMIM 607196* | | | | | |
| Kelley,  2002  USA^47^ | Inf  1 | Disorders with 2-ketoglutaric aciduria Mutation | Amish phenotype, with 2-ketoglutaric aciduria; lethal outcome. | organic acid  lactates  Brain MRI, gene sequencing | NC |
| Dunckelmann,  2000  Germany^51^ | Inf  1 | Neuro manifestation of 2-ketoglutarate dehydrogenase deficiency with 2-ketoglutaric aciduria. | crisis of episthotonus, hyperexcitability and truncal hypertonia, which became less pronounced at age of 14 months | organic acid  2-KGD activity in skin fibroblast  Brain MRI | NC |
| Rajab,  2007  Oman^48^ | Inf  4 | A familial form of lethal microcephaly with simplified gyral pattern and brain stem hypoplasia | diagnosed in prenatal period with low birth-weight and length, facial features of Amish microcephaly; at birth he presented with respiratory distress, hypoventilation, apnea, seizures, followed by death | Brain MRI  genetic sequencing | NC |
| Spiegel,  2009  Israel^49^ | Child 2  Ado 2 | *SLC25A19* mutation as a cause of neuropathy and bilateral striatal necrosis. | recurrent flaccid paralysis, encephalopathy, chronic progressive polyneuropathy | genetic sequencing | NC |
| Siu,  2010  Canada^50^ | Child  1 | Long-term survival in a patient from consanguineous Amish parents | antenatal microcephaly, postnatal facial Amish phenotype; presented with a neurological crisis responding to a high fat diet; survived till 7 yr of age with development delay | organic Acid  lactates  Brain MRI | NC  fat diet |
| Genetic defects in thiamine pyrophosphokinase (**TPK) 1** or THMD5 that block the conversion of free thiamine in the cell to thiamine diphosphate  *(THMD5) OMIM 60637* | | | | | |
| Mayr,  2011  Austria^32^ | Inf  2  Child  3 | Encephalopathic children with defects in the pyruvate oxidation pathway,  from non or consanguines Iraqui parents | 2 patients: developmental delay progressing to encephalopathy; 3 other patients: initial normal development progressing to either ataxia, disturbed gait, truncal ataxia, upper limb dystonia; all died | lactate  organic acid  Brain MRI  muscle biopsy | NC |
| Huang,  2019  China^52^ | Child  1 | [Reduced thiamine binding: a cause of TPK deficiency](https://www.ncbi.nlm.nih.gov/pubmed/30483896) with a TPK1 mutation | episodic encephalopathy | ETK-AC  genetic sequencing | + |
| Nyhan,  2019  USA^53^ | Child  1 | TD [and mutation in TPK1 resembling BGTGD](https://www.ncbi.nlm.nih.gov/pubmed/31404531) | Leigh syndrome phenotype; B1 + biotin did not stop the course of the disease | serum biotin  genetic sequencing | NC |
| [Eckenweiler](http://orcid.org/0000-0003-2461-961X),  2020  Germany^54^ | Inf  1 | B1 Treatment and Favorable Outcome of a Biallelic TPK1 Variant | acute encephalopathy, progressive onset of neuro dysfunction, ataxia, dystonia, spasticity | genetic sequencing | +  biotin |
| **C2 - TD in diabetic and/or obese patients** | | | | | |
| Clark,  2006  USA^55^ | Ado  1 | acute TD in diabetic ketoacidosis: diagnosis and management | hyperglycemia, diabetic ketoacidosis, then persistent encephalopathy, clinical improvement with B1 administration and osmotherapy | serum thiamine  blood gas Brain CT | + |
| Rosner,  2015  USA^56^ | Child 2  Ado  18 | low B1 level in pediatric type 1 diabetes and keto-acidosis | diabetes with diabetic ketoacidosis encephalopathy; 35% with low thiamine status and responsive to B1 challenge | serum thiamine | + |
| **C3 - TD after bariatric surgery** | | | | | |
| Armstrong  -Javors,  2016  USA^59^ | Ado  1 | WE in adolescents after bariatric Roux-en-Y  TPN unsupplemented in B1 | classic WE, confirmed thiamine assay,  effective treatment challenge | serum thiamine  normal MRI | + |
| Towbin,  2004  USA^57^ | Ado  3 | Beriberi after laparoscopic Roux-en-Y gastric bypass in obese adolescence. | in the post-operative period: nausea and vomiting (treated with ranitidine); later developed signs of dry beriberi or truncated WE with confirmed multiple micronutrient deficiencies without abnormal CRP | serum thiamine  EMG  Brain CT | + |
| Stenerson,  2013  USA^58^ | Ado  1 | Recurrent WE in an adolescent post gastric bypass; multivitamin removed | morbid obesity (body mass index of 54.3 kg/m^2^), developed WE signs whenever the patient was not compliant to multivitamin therapy | serum thiamin  Brain MRI | + |
| Samanta,  2015  USA^60^ | Ado  1 | Dry beriberi preceded WE post sleeve gastrectomy; vitamin removed | post-operative nausea treated with omeprazole metoclopramide, followed by dry beriberi (lower limb numbness, walk required assistance) and WE; B1 reversed ocular signs but not neuropathy | serum thiamine  CRP  Brain MRI | + |
| Takacs,  2018  USA^61^ | Ado  1 | Ophthalmoplegia and pediatric WE after Roux-en-Y gastric surgery + IV fluid without B1 | patient submitted to bariatric surgery for morbid obesity associated with Type 2 diabetes; an inappropriate thiamine supply in the post-operative period resulted in a classic WE (notably altered mental status and gait ataxia) which improved by B1 supply | serum thiamine  Brain MRI, CT | + |
| **C4 - TD related to parenteral or enteral nutrition** | | | | | |
| Thauvin-Robinet,  2004  France^62^ | N  11 | Lactic acidosis and acute TD a report of neonates with unsupplemented TPN | onset of hyperperlactatemia, metabolic acidosis, delay in B1 supplementation was associated with death | blood  lactate | + |
| Masumoto,  2009^63^  Japan | Child  8 | Thiamine trends in post abdominal surgery starvation period without B1 supply. | gradual decrease of B1 level during post abdominal surgery starvation period in patients on TPN without B1 supplementation. | whole blood TDP | + |
| Ramsi,  2014  USA^64^ | Ado  1 | Lactic acidosis and multi-organ failure due to TD during B1- deficient TPN post colectomy | WE + Cardiac failure + hypotension with multi-organ failure  refractory lactic acidosis was reversed by B1 | serum thiamine  serial lactates  TT Echo | + |
| Long,  2014  China^65^ | Ado  1 | WE and Wet beriberi secondary to unsupplemented TPN in post abdominal surgery | WE and cardiac beriberi preceded by non specific abdominal symptoms and treated with aripiprazole; empiric thiamine challenge was effective | Brain MRI FLAIR  lactates | + |
| Benidir,  2014  Canada^68^ | Child  1 | Visual abnormalities in B1 unsupplemented TPN child with liver transplant and short bowel syndrome. | Truncated WE, initial serum thiamine undetectable; empiric B1 challenge improved mental status | serum thiamine  CRP  Brain MRI | + |
| Salvatori,  2016  Italy^21^ | N  2 | Thiamine deficiency in a developed country: acute lactic acidosis in neonates due to unsupplemented TPN | 2 preterm infants with < 32 weeks of gestational age, cardiac dysfunction and refractory lactic acidosis; both with heart dysfunctions reversed with B1 | serum thiamine  lactates  organic acid  TTEcho | + |
| Lefevre,  2018  USA^66^ | Ado  1 | Myocardial ischemia in TD secondary to TPN suspension due to allergic reaction | wet beriberi followed by truncated WE  reversed with B1 | serum thiamine  lactates  TTEcho | + |
| **C5** **– TD related to high consumption of sugar-sweetened beverages** | | | | | |
| Nozue,  2001  Japan^69^ | child  1 | Water intoxication and lactic acidosis caused by excessive intake of commercial sports drink | progressive metabolic acidosis;  TD associated hyperlactatemia | lactates |  |
| Takemoto,  2008  Japan^71^ | Inf  1 | Infantile beriberi caused by excessive intake of isotonic drink | classic infantile beriberi | NA |  |
| Saeki,  2010  Japan^22^ | Inf  2 | WE due to excessive intake of isotonic drink or overstrict diet therapy in Japanese children. | 1: excessive isotonic drinks, vomiting somnolence; WE  2: WE secondary to restricted diet in early infancy and excess sport drinks, with hyperlactatemia (serum, CSF) | serum thiamine  lactates  Brain MRI | + |
| Ko,  2011  China^78^ | Ado  1 | WE associated with a “high risk lifestyle”: junk food, binge drinking, ketamine use, smoking habits | acute WE, B1 supplementation resulted in recovery of mood and memory | ETK-AC  lactates  TTEcho  Brain CT, MRI | + |
| Kataoka,  2012  Japan^74^ | Inf  1 | B1 reversible severe pulmonary artery hypertension secondary to excessive intake of isotonic drink. | dry and wet beriberi with pulmonary arterial hypertension | serum thiamine  TTEcho | + |
| Fujii  2012,  Japan^73^ | Inf  1 | Infantile wet beriberi,  regular isotonic drinks, undernutrition | wet beriberi and truncated WE, dysfunction of left and right heart ventricules and pericardial effusion. | lactates  Brain MRI  TTEcho, ECG | + |
| Majima,  2013  Japan^70^ | Child  1 | Use of central venous saturation monitoring in a pediatric cardiac beriberi secondary to prolonged  consumption of isotonic drink | initial suspicion of myocarditis, then ruled out; evidence of high output cardiac failure with high, central venous Scv02 and hyperlactatemia, cardiac beriberi, positive thiamine challenge ensues no sequelae | serum thiamine TTecho  lactates  organic acids  central venous SO2 | + |
| Hiraki,  2014  Japan^76^ | Inf  2 | WE cases due to excessive intake of isotonic drink and unbalanced diets | vomiting followed by either Classic or truncated WE and seizures; both improved with B1 but sequelae with mental retardation observed in patient 2 | serum thiamine  Brain MRI | + |
| Shioda,  2014  Japan^75^ | Child  1 | A case of pediatric WE due to excessive intake of isotonic drinks | WE precipitated with intravenous fluids containing glucose but no vitamins, recovery with B1 without neurological sequelae | serum thiamine  Brain MRI | + |
| Okumura,  2018  Japan^77^ | Inf  28  Child  5 | Children with prolonged regular soft drink, with a subset of poor food intake and unbalanced diet (polished rice) | vomiting, lethargy, then WE and wet beriberi, initial low thiamine level, and metabolic lactic acidosis, variable outcome after thiamine supplementation (death, ocular and cognitive disorders, motor impairment, swallowing disorder, autism, epilepsy) | whole blood  TDP  🡽 lactate  TT echo  Brain MRI | NC |
| Sakurai,  2019  Japan^72^ | Inf  2 | Pulmonary arterial hypertension induced by TD as a result of excessive soft drink consumption and unbalanced diet (polished rice) | 1) wet beriberi with pulmonary arterial hypertension unresponsive to nitric oxide, following initial episode of diarrhea and reversed with B1  2) dry and wet beriberi with pulmonary arterial hypertension reversed with B1 | whole blood  TDP  TT Echo  -color doppler | + |
| **C6 - TD related to eating disorders (anorexia)** | | | | | |
| Winston,  2000  UK^79^ | Ado  21 | Prevalence of biological TD in pediatric anorexia nervosa | cohort study of 37 attendees of an anorexia nervosa Specialized Center, 14 patients (38%) within deficiency range; 7 (19%) within stringent criterion for deficiency,  overlapping neuropsychiatric symptoms | ETK-AC |  |
| Heiser,  2004  Germany^80^ | Ado  1 | neuropathy due to hypovitaminosis after excessive weight loss | patient presenting an eating disorder ( anorexia, refusal to feed, vomiting, weight loss), developed dry beriberi with polyneuropathy, associated with multivitamin deficiencies | serum thiamine  other vitamins  EMG | + |
| Peters,  2007  USA^81^ | Ado  1 | WE in a patient with anorexia nervosa of restricting type, strict diet depleted in B1 + SSB binge drinking | weight loss, confusion, ataxia, nystagmus (WE) | Brain MRI | + |
| French,  2013  USA^84^ | Ado  1 | ascending weakness in adolescent with strict restricted diet for weight loss, oppositional behaviour | patient with calf pain, tripping, and progressive inability to walk., neuropathy secondary to hypovitaminosis | serum thiamine  ETK-AC | + |
| Renthal, 2014  USA^82^ | Ado  1 | peripheral neuropathy and WE in adolescent with anorexia nervosa | dry beriberi (peripheral polyneuropathy) and truncated WE with preserved mental status | serum thiamine  Brain MRI  EMG | NA |
| Bahat,  2020  Israel^83^ | Ado  4 | Thiamine status in 69 anorexic patients: 44 r estrictive type and 25 binge-eating/purging | four anorexic girls, 3 in eating/purging type, and 1 in restrictive type out of 39 nutritional management, naive group had lower TDP | whole blood TDP | + |
| **C7 - TD related to gastrointestinal disorders** | | | | | |
| Rodan,  2013  Canada^91^ | Child  1 | MR spectroscopy in pediatric WE, post abdominal surgery and short gut syndrome | classic WE: based on clinical, biochemical, and MRI findings | serum thiamine  lactates  Brain MRI, MRS | + |
| Barnes  2016,  USA^86^ | Ado  1 | TPN dependent teenager with complicated colectomy for ulcerative Colitis on infliximab, steroids; lack of B1 in TPN | truncated WE associated with refractory hyperlactatemia and normal CRP | serum thiamine  lactates | + |
| Salloum,  2018  USA^87^ | Ado  1 | Beriberi disease following total colectomy for Hirschsprung disease, under loperamide, metronidazole, 1 week a month, for suspected small intestinal bacterial overgrowth | dry beriberi; with ataxia, unsteady gait, and weakness leading to inability to walk.  Normal CRP and erythrocyte sedimentation rate | serum thiamine  Brain MRI  EMG | + |
| Anderson,  2020  USA^90^ | Ado  1 | TD Type B lactic acidosis in a patient with Crohn’s disease following subtotal colectomy and unsupplemented TPN | persistent refractory type B lactic acidosis. | lactates | + |
| Greenspon,  2010  USA^85^ | Child  1 | Shoshin beriberi mimicking central line sepsis in a child with short bowel syndrome receiving unsupplemented TPN (B1), antibiotics, metronidazole | shock progressing to severe hyperlactatemia, low CRP (1.7 mg/dL), negative blood culture, blood sample obtained prior to thiamine challenge was lost. | lactates  TTecho | + |
| Roilides,  2019  Greece^88^ | Child  1 | TD in a short Bowel Syndrome.  TPN dependent with recurrent sepsis, malnutrition and small intestinal bacterial overgrowth: cyclic supply of rifaximin and probiotics | WE secondary to multiple factors following an episode of vomiting, positive response to thiamine challenge and biochemical TD confirmed 17 days later | Brain MRI | + |
| Zhang,  2020  China^89^ | N  1 | TD tied to short bowel syndrome in a TPN dependent extremely low birth weight (1.258 Kg) preterm infant (29 weeks) | WE with low thiamine level and normal CRP, clinical improvement with B1 but neurodevelopmental sequelae (speech, hand-eye coordination, social interactions) | serum thiamine  Brain CT, MRI | + |
| **C8 - TD in pediatric malignancies** | | | | | |
| D'Aprile,  2000  Italy^92^ | Ado  1 | WE associated with TPN in patient with acute leukemia | WE, unusual bilateral brain lesions, reversed under B1 therapy evidenced by serial MRI studies | Serial Brain MRI | + |
| Bae,  2001  South Korea^104^ | Ado  1 | WE in a child with acute myeloblastic leukemia associated with vomiting and post-chemotherapy anorexia | WE onset at 30 days post-chemotherapy | serum thiamine  Brain MRI | + |
| Takahashi,  2013  Japan^102^ | Ado  1 | WE in a 21-trisomy girl with acute lymphoblastic leukemia | WE responding to high dose of thiamine without sequelae | serum thiamine  Brain MRI | + |
| Perko,  2012  USA^105^ | Child  4  Ado  1 | Cases of pediatric WE tied to TPN without multivitamin (nationwide shortage) | Truncated or classic WE + hyperlactatemia:  1) ataxia, ocular signs in an irradiated patient with medulloblastoma  2) altered speech: osteosarcoma on methotrexate  3) metabolic acidosis in an irradiated patient with rhabdomyosarcoma  4) WE: in a patient with medulloblastoma ( chemotherapy, autologous stem-cell rescue) fatal outcome.  5) WE in an acute myelogenous leukemia patient | lactates  Brian MRI, CT  EEG | + |
| Park,  2014  S. Korea^106^ | Ado  1 | WE in child with neuroblastoma requiring high dose of thiamine on poor oral diet and prolonged vomiting | truncated WE in a patient on chemotherapy with auto peripheral stem cell transplant responding to high dose of thiamine | serum thiamine  Brain MRI  FLAIR - EEG | + |
| Darlington,  2015  USA^107^ | Child  1 | Stem cell transplant-associated WE in a patient with neuroblastoma (stage 4) | facial rash and truncated WE  with micronutrient deficiencies  (vitamins E, A, B1, niacin, folate, zinc) | serum thiamine  Brain MRI | + |
| Cefalo,  2014  Italy^94^ | Child  2 | WE reversible with thiamine in pediatric neuro-oncology | WE during the management of a brain tumor | serum thiamine  Brain MRI, CT | + |
| Svahn,  2003  Italy^93^ | Inf  1 | Lactic acidosis due to TD in a patient with B-cell **l**eukemia /lymphoma on TPN during high-dose methotrexate | lethargy with type B lactic acidosis refractory to bicarbonate, reversed by B1 therapy | lactates | + |
| La Spina,  2010  Italy^95^ | Ado  1 | WE post-chemotherapy of acute myeloid leukemia (FAD M5) in teenager on TPN formula without multi vitamin supplement for mucositis | WE: ocular signs, auditory hallucinations, altered consciousness, drowsiness, unsteady gait (secondary to poor intake and vomiting under chemotherapy) | Brain MRI | + |
| Zensho,  2018  Japan^96^ | Child  1 | WE in a child with autism and T-cell acute leukemia on chemotherapy with poor oral dietary and vomiting | post-chemotherapy WE (prednisolone, daunorubicin dexamethasone, vincristine, L-asparaginase), responded to high dose B1 | serum thiamine  Brain MRI, CT | + |
| Didisheim,  2020  Switzerland^97^ | Ado  1 | TD lactic acidosis in critically ill teenager, with chemotherapy for leukemia and under TPN | refractory shock requiring ECMO, persistent hyperlactatemia, normal sepsis work-up | serial lactates | + |
| Pediatric Stem Cell Transplantation and polypharmacy | | | | | |
| Byun,  2007  South Korea^100^ | Ado  1 | Type B lactic acidosis due to TD tied to lack of B1 in TPN and poor intake in acute lymphoblastic leukemia and bone marrow transplantation | shock with hyperlactatemia | serial lactates | + |
| Han,  2012  South Korea^101^ | Child  1  Ado  1 | WE receiving hemato-stem cell transplant: irradiated body, tacrolimus, cyclophosphamide, methotrexate, metronidazole, cyclosporine | patient 1: truncated WE, low oral intake on TPN, partial reversal on B1 then death (septic shock)  patient 2: WE, vomiting, patient under TPN . | serum thiamine  Brain MRI | + |
| Lerner,  2017  Israel^103^ | Child  2 | Lactic acidosis in TD children with bone marrow transplantation for leukemia and burkitt lymphoma | patient 1: myelomonocytic leukemia, diarrhea, hyperlactatemia  patient 2: Burkitt's lymphoma, truncated WE  associated type B hyperlactatemia | serum thiamine  ETK-AC lactates,  TTEcho,  Brain CT | + |
| Derespina,  2018  USA^98^ | Inf  1 | Lactic acidosis due to TD on cyclic chemotherapy for autologous stem cell transplantation and TPN | mucositis, metabolic acidosis with hyperlactatemia, confirmed TD (with laboratory result delay) | serum thiamine  Lactates | + |
| Murali,  2020  USA^99^ | Inf  1 | Persistent lactic acidosis in an infant with myelomonocytic leukemia and receiving bone marrow transplant, on multivitamin TPN but developed an allergic reaction to intravenous fat emulsion, and gut graft versus host disease | sustained hyperlactatemia, hyperpyruvatemia  multivitamin deficiencies (B1, 2, 3, 6, 9, Vit C D) | serum thiamine  Lactates  organic acid | + |
| **C 9 - TD in critically ill patients** | | | | | |
| Pediatric dialytic unit of nephrology | | | | | |
| Pela,  2000  Italy^121^ | Child 1 | Hemodiafiltration efficacy in a lactic acidotic child due to TD | hyperammonemia and severe metabolic lactic acidosis | serum thiamine  Lactates | + |
| Baracco,  2013  USA^126^ | Ado  1 | Acute WE in TPN malnourished on end renal lupus disease, following peritoneal dialysis | truncated WE (altered motor and consciousness), with evidence of low B1 and B12 | serum thiamine  TTEcho  MRI | + |
| Harshman,  2018  Germany^112^ | Ado  1 | Micronutrients (B1) deficiencies in pediatric dialysis patient receiving bone marrow transplant and TPN due to gut graft-versus-host disease | WE secondary to TPN inadvertently lacking for B1, hyperlactatemia | Lactates | + |
| Elias,  2019  Canada^114^ | Ado  1 | Shoshin beriberi post-kidney transplant with rapid recovery after B1 administration | fulminant heart failure associated with, hyperglycemia, metabolic acidosis, hyperlactatemia | Lactates  TTEcho | + |
| Pediatric cardiology | | | | | |
| Sakuma,  2013  Japan^125^ | Child  1 | TD Induced Pulmonary Hypertension in an Infant with unbalanced diet consisting in monotonous rice staple | truncated WE, dry and wet beriberi, with heart failure and pulmonary arterial hypertension | serum thiamine  TTEcho | + |
| Cottini,  2016  Italy^110^ | Ado  1 | Shoshin Beriberi and accidental hypothermia as causes of heart failure in a malnourished child while refeeding | wet beriberi, shoshin with cardiogenic shock and worsening lactic acidosis | Lactates  TTEcho | + |
| Vicinanza, 2019  Belgium^113^ | Child  1 | Shoshin Beriberi and accidental hypothermia as causes of heart failure in a malnourished child while refeeding | heart failure on refeeding in documented multiple micronutrient deficiencies | Serial lactates  TTEcho, ECG  Brain MRI | + |
| Shamir,  2000  Israel^119^ | Child  10 | Biochemical TD in children with congenital heart disease before and after corrective surgery | pre-cardiac surgery: 4 children were detected as thiamine deficient  post-cardiac surgery: 6 children showed gradual decline in serum thiamine after surgery | ETK-AC | + |
| Miscellaneous pediatric critical conditions | | | | | |
| Teagarden,  2017  USA^111^ | N  1 | TD leading to refractory lactic acidosis and pulmonary arterial hypertension in a pediatric patient following an unsupplemented TPN | pulmonary arterial hypertension, with hemodynamic instability, persistent refractory lactic acidosis, no CRP assay available,  effective thiamine challenge | serum thiamine  lactates  work up sepsis  TTEcho | + |
| Weiss,  2019  USA^115^ | Child  6 | Matched retrospective cohort study of thiamine to treat persistent hyperlactatemia in pediatric septic shock | septic children with low thiamine, hyperlactatemia and no CRP assay; two outcomes: lactate time pattern T0, T24, T48, T72 (outcome I) and clinical markers of critical illness (outcome II); B1 challenge improved only outcomes I | serum thiamine  serial lactate  blood gas  PELOD-2  Prism II | + |
| Cooke,  2006  UK^123^ | Ado  1 | Atypical WE in a teenager: ocular signs as predominant presentation, malnutrition, selective diet for fear of choking recurrence | truncated WE following recurrent vomiting, poor appetite, weight loss, bilateral blurred vision as central haziness, nystagmus upbeat in the primary position with right and left gaze paresis, lethargy and unsteady gait | serum thiamine  Lactates  Brain CT, MRI | + |
| Pediatric neurology | | | | | |
| Decker,  2000  USA^108^ | Child  1 | TD and altered mental status after prolonged starvation | WE | Brain MRI | + |
| Coe, 2001  Italy^118^ | Child  1 | WE in a child: case report and MR findings preceded by context of food refusal and soft drinks | MRI revealed WE unsuspected clinically (the patient presenting with lethargy); B1 clinical improvement with residual mild memory deficit | EEG  Brain MRI  gadolinium | + |
| Lamdhade,  2014  Kuwait^23^ | Ado  1 | Recurrent WE with atypical clinical and radiological feature in an adolescent suffering from congenital intestinal atresia on TPN | recurrent vomiting, failure to thrive and no compliance to dietary supplement, the patient received IV fluids only.  1st episode: one week later, while fully conscious the patient developed truncated WE  2nd episode: recurrent vomiting led to truncated WE | serum thiamine  Brain CT, MRI  FLAIR | + |
| PTD caused by unbalanced diets | | | | | |
| Kesler,  2005  Israel^122^ | Child  3 | Ophthalmoplegia and nystagmus in infants fed a thiamine-deficient soy- based formula: resulting from an epidemic of WE in Israel | truncated WE with hyperlactatemia (gaze abnormalities nystagmus, abnormal eye movements, apathy, vomiting, convulsions) | ETK-AC  Lactates (serum, CSF)  Brain CT, MRI | + |
| Abu-Kishk,  2009  Israel^124^ | Inf  2 | Infantile WE due to vitamin deficiency in industrial countries; strict vegetarian mother precipitated by intravenous glucose fluids | truncated WE associated with megaloblastic anemia; confirmed avitaminosis of B1, B12  methylmalonic aciduria | serum thiamine  ETK-AC  lactates, organic acid ,  Brain MRI,MRS | + |
| Kubota,  2015  Japan^117^ | Child  1 | WE due to a severely unbalanced diet (polished rice, noodles) in young patient | initially diagnosed as Guillain-Barré syndrome later rectified as dry beriberi (neuropathy) then onset of WE, hyperlactatemia, normal CRP, EMG | serum thiamine  lactates,  acid organic  EMG  Brain MRI | + |
| Ahmed,  2012  Saudi  Arabia^120^ | Ado  1 | Impact of prolonged Ramadan period | following Ramadan fasting period, the patient presented with repeated vomiting, abdominal discomfort, and truncated WE (lethargy, confusion, drowsiness, diminished vision) | serum thiamine  lactates  Brain MRI | + |
| Quatresous, 2014  France^127^ | Inf  32 | Epidemic Beriberi in Mayotte; infants breastfed by thiamine deficient mothers (pre/postpartum consuming rice bouillis (oubou) with neuropathy and poor socio economic status | initial refusal of food, rhinorrhea, vomiting, and soft stools; progressively suffered from afebrile respiratory distress, hepatomegaly, metabolic acidosis with high CRP; thereafter, patients frankly presented with wet beriberi, ascites, pericarditis, pulmonary arterial hypertension, collapsus and cardiac failure.  B1 reversed the symptoms except for subset with delayed diagnostic | serum thiamine  ETK-AC  x chest Ray  TTEcho | +  if timely supplied |
| Moulin,  2014  France^109^ | Inf  1 | Infant exclusively breastfed by thiamine deficient mother | after an episode of diarrhea and vomiting, the patient developed high output heart failure (cardiomegaly), metabolic acidosis, with hyperlactatemia, normal CRP | serum thiamine  Whole blood TDP  lactates, TTEcho | + |
| Tiwana,  2020  USA^116^ | Ado  1 | Novel form of WE retinal hemorrhage in a pediatric patient on unbalanced diet | truncated WE (progressive confusion, impaired memory recall/repetition, ocular abnormalities, vertical nystagmus) | Brain MRI, FLAIR  funduscopy | + |
| **C 10 - TD associated with autism spectrum/botulism** | | | | | |
| Duvall,  2013  USA^131^ | Child  1 | Vitamin deficiencies (C, B1) in an autistic child with restricted diet | autism spectrum disorder with dry beriberi (limb pain) and wet beriberi (pulmonary arterial hypertension); multivitamin deficiencies (B1, B6, B12, C, D) | serum thiamine,  TTEcho | + |
| Baird,  2015  USA^129^ | Ado  1 | Vitamin B deficiencies in a critically ill autistic child with a restricted diet | autism spectrum disorder with altered consciousness, status epilepticus  severe lactic acidosis, with liver dysfunction | serum thiamine  Lactates | + |
| Lonsdale,  2002  USA^132^ | Child  3 | Children with autism spectrum disorder [treated with thiamine tetrahy-drofurfuryl disulfide](http://www.nel.edu/pdf_w/23_4/NEL230402A02_Lonsdale_rw.pdf) | overlapping signs of TD and autism spectrum; beneficial effect of thiamine tetra-hydrofurfury disulfide | serum thiamine  ETK-AC | + |
| Ringe,  2014  Germany^128^ | Inf  1 | [TD](http://pediatrics.aappublications.org/content/134/5/e1436) secondary to thiaminase producing clostridium botulinum serotype A2 infection | signs of botulism with respiratory failure and faster improvement after thiamine therapy | microbio feces (thiaminase +) | + |
| Watanabe,  2009  Japan^130^ | Inf  1 | Infantile autism with eating disorder; on TPN (unsupplemented in B1) | truncated WE | Brain MRI  FLAIR | + |

**Legend:**

For age range: N neonate; Inf: infant; Child: childhood; Ado: adolescence.

For response to thiamine: NA: not available; NC: not clear; **+**: positive (clinical or lactates or neuroimaging)

B1: vitamin B1 (thiamine)

Brain CT: Brain computerized tomography

BTBGD: Biotin–thiamine-responsive basal ganglia disease

CT: computerized tomography

CSF: cerebrospinal fluid ,

EMG: electromyography

ETK-AC: erythrocyte transketolase activity coefficient

ECMO: extracorporeal membrane oxygenation

FLAIR: Fluid-attenuated inversion recovery

MRI: magnetic resonance imaging

PELOD-2: Pediatric Logistic Organ Dysfunction-2 score

TD: Thiamine deficiency

TDP: Thiamine diphosphate

TPK: Thiamine pyrophosphokinase;

TRMA: Thiamine-responsive megaloblastic anemia

TPN: total parenteral nutrition

TTEcho: transthoracic echocardiography doppler

WE: Wernicke's encephalopathy

lactates: assessment of lactate levels
